# Necrotizing Fasciitis by Extended-Spectrum Beta-Lactamase-Producing Escherichia coli as the Initial Presentation of Hairy Cell Leukemia: A Case Report

**DOI:** 10.7759/cureus.88614

**Published:** 2025-07-23

**Authors:** Diego Pérez-Valdez, Raúl Rodolfo Sánchez-Rosado, Lucero Janeth Ortiz-Pacheco, Rodrigo Villarreal-Zavala, Samuel Hernández Alvarado

**Affiliations:** 1 General Surgery, Hospital General Regional No. 1 “Ignacio García Téllez”, Instituto Mexicano del Seguro Social (IMSS), Mérida, MEX; 2 Colon and Rectal Surgery, Hospital General de México, Mexico City, MEX

**Keywords:** colorectal surgery, hairy cell leukemia, hematologic malignancy, necrotizing fascitis, perianal infection, septic shock, surgical debridement

## Abstract

Necrotizing fasciitis (NF) is a rapidly progressive and life-threatening soft tissue infection. Fournier’s gangrene (FG), a subtype involving the perianal and genital regions, is especially severe in immunocompromised individuals. Although rare, hairy cell leukemia (HCL) can initially present with severe infections due to profound immunosuppression. We report the case of a 53-year-old man with hypertension who developed a perianal abscess caused by extended-spectrum beta-lactamase (ESBL)-producing *Escherichia coli*. Despite surgical drainage and carbapenem therapy, the infection progressed. Laboratory studies revealed pancytopenia, and bone marrow biopsy confirmed HCL with 95% infiltration. Despite initiating rituximab, the patient’s condition deteriorated. Imaging showed a large presacral abscess, rectal wall perforation, and perirectal emphysema. Surgical management included diverting loop colostomy, extensive debridement, and abdominal lavage. Postoperatively, the patient developed multiorgan failure and died of septic shock despite intensive care. This case underscores the diagnostic and therapeutic challenges of FG in patients with hematologic malignancies. HCL can present with profound immunosuppression, predisposing to severe, monomicrobial NF caused by multidrug-resistant organisms. ESBL-producing* E. coli *further complicates management. Imaging is essential to assess disease extent, and early surgical intervention is critical. Delay in diagnosing the underlying malignancy and the immunocompromised state contributed to a fatal outcome. FG may be the initial manifestation of occult hematologic malignancy such as HCL. Clinicians should maintain a high index of suspicion for immunosuppressive disorders in patients with severe infections and pancytopenia. Multidisciplinary management, including prompt debridement, broad-spectrum antibiotics, and early hematologic evaluation, is vital to improve outcomes.

## Introduction

Necrotizing fasciitis (NF) is a rare but rapidly progressive soft tissue infection characterized by extensive fascial necrosis and systemic toxicity. Among its subtypes, Fournier’s gangrene (FG) primarily affects the perineal and genital regions and is associated with high morbidity and mortality, particularly in immunocompromised patients [1-3]. Initial symptoms may include swelling, pain, and fever, but progression to sepsis and multiorgan failure can occur rapidly if not promptly recognized. Early clinical signs may be nonspecific, often leading to delayed diagnosis, systemic deterioration, and septic shock [1].

FG is classically polymicrobial (type I NF); however, monomicrobial infections (types II and III) caused by Gram-negative bacilli, particularly *Escherichia coli*, have been increasingly reported [2,4]. Of particular concern is the emergence of extended-spectrum beta-lactamase (ESBL)-producing *E. coli*, which confer resistance to most beta-lactam antibiotics, including penicillins and cephalosporins. These multidrug-resistant strains significantly complicate antimicrobial therapy and are associated with worse clinical outcomes [3-5]. Empiric treatment should therefore include carbapenems, with subsequent de-escalation based on culture results [3].

Hairy cell leukemia (HCL) is an uncommon indolent B-cell lymphoproliferative disorder accounting for approximately 2% of all leukemias [1,6]. It is characterized by profound immunosuppression resulting from monocytopenia, neutropenia, and splenic sequestration, which predispose patients to opportunistic and severe bacterial infections [1,4]. Although HCL is typically diagnosed during routine blood testing or for cytopenia-related symptoms, in rare cases, life-threatening infections may be the first clinical sign of underlying malignancy. In some cases, life-threatening infections may precede or lead to the diagnosis of HCL [6].

Although rare, the occurrence of FG as the initial manifestation of undiagnosed HCL presents a critical diagnostic and therapeutic challenge. We report the case of a 53-year-old male who developed perianal NF caused by ESBL-producing *E. coli *as the first clinical manifestation of HCL. This case underscores the importance of maintaining a high index of suspicion for underlying hematologic malignancies in patients with severe soft tissue infections and unexplained pancytopenia. Prompt surgical, microbiological, and hematologic evaluation is essential to improve patient outcomes.

## Case presentation

A 53-year-old male with a medical history of systemic arterial hypertension, managed with lecarnidipine and nebivolol and no history of immunosuppressive therapy, presented in February 2024 with painful swelling in the perianal region. He reported progressive pain, foul-smelling discharge, and low-grade fever. Initial management at a private facility included surgical drainage of a perianal abscess on March 4, 2024. Intraoperative cultures identified ESBL-producing *E. coli*, prompting initiation of meropenem and amikacin.

Despite appropriate antimicrobial therapy, the wound failed to improve clinically. At the time of clinical deterioration, the patient was febrile (38.6°C), tachycardic (heart rate: 112 bpm), and hypotensive (BP: 92/56 mmHg) and had oxygen saturation of 93% on room air.

On March 8, 2024, four days after initial drainage and approximately two weeks from symptom onset, laboratory results revealed severe pancytopenia: leukocytes 0.51 × 10⁹/L, neutrophils 0.16 × 10⁹/L, hemoglobin 6.0 g/dL, and platelets 30 × 10⁹/L. C-reactive protein and procalcitonin were elevated (CRP: 310 mg/L; PCT: 21.3 ng/mL), suggesting ongoing systemic inflammation and sepsis. A diagnostic workup was initiated. Bone marrow biopsy confirmed HCL, with 95% infiltration by abnormal B-lymphocytes positive for CD20, tartrate-resistant acid phosphatase (TRAP), and annexin A1.

**Figure 1:**
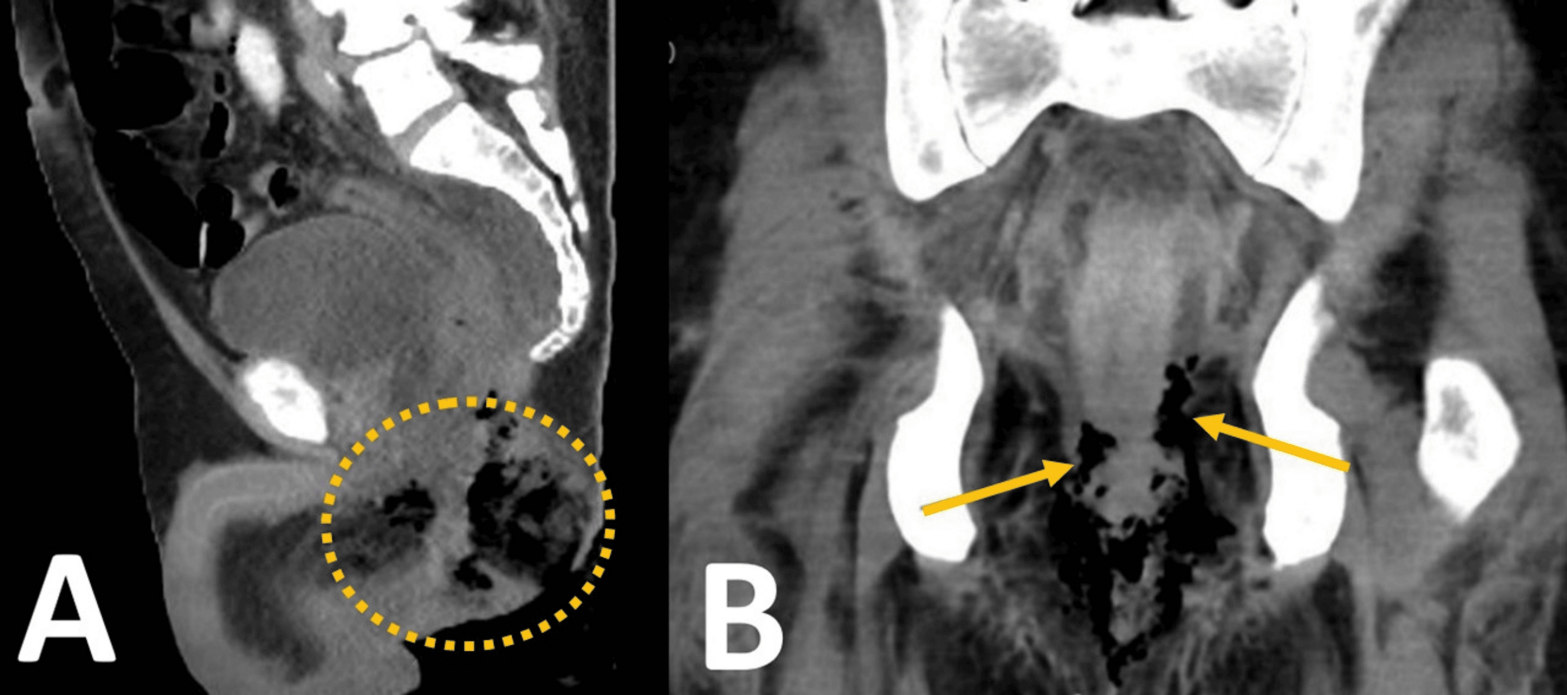
CT findings in this case of Fournier’s gangrene. (A) Sagittal view shows perirectal soft tissue gas and a large presacral fluid collection (green circle). (B) Coronal view reveals bilateral gas tracking along the ischiorectal fossae (green arrows), consistent with necrotizing infection.

While sepsis can induce transient marrow suppression, the severity and persistence of pancytopenia, along with diagnostic marrow infiltration by clonal B-cells, confirmed underlying HCL. The patient received the first dose of rituximab on March 22, 2024 - 14 days after pancytopenia was first detected. The perianal wound continued to deteriorate, with expanding necrosis and persistent purulent, non-fetid exudate. By late March, the patient developed diffuse abdominal discomfort. A contrast-enhanced computed tomography (CT) scan performed on March 31 revealed rectal wall thickening, perirectal emphysema, and a sizable presacral fluid collection measuring 51 × 37 × 90 mm (estimated volume 88.8 mL), along with suspected perforation of the left lateral rectal wall (Figure 1).

**Figure 2:**
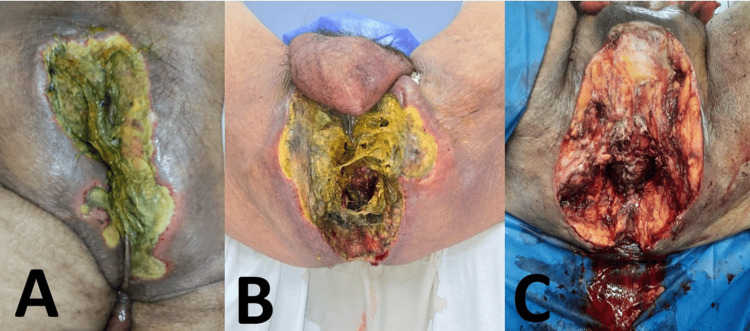
Clinical evolution of perianal necrotizing fasciitis. (A) Initial presentation with perianal necrosis and greenish purulent discharge. (B) Progressive necrosis and extensive tissue sloughing. (C) Post-debridement image showing exposed fascia and complete involvement of both ischiorectal fossae.

Due to the patient's clinical deterioration and imaging findings, surgical exploration was performed on April 15, 2024. The procedure included a diverting loop colostomy, extensive debridement of necrotic perianal and ischiorectal tissues, and abdominal lavage. Intraoperative findings included a 20 mm perforation in the left lateral rectal wall, necrosis of the mesorectum, and full involvement of the ischiorectal fossae. Hemostasis was particularly challenging due to severe thrombocytopenia. The post-debridement surgical field revealed extensive fascial involvement and exposed soft tissues across the ischiorectal fossae, as illustrated in Figure 2.

Postoperatively, the patient required vasopressor support and was admitted to the intensive care unit (ICU).

Despite maximal supportive care, including broad-spectrum antimicrobials, blood product transfusions, and critical care measures, the patient’s condition progressively worsened. He ultimately died of septic shock on April 21, 2024. A chronological summary of the clinical events, interventions, and outcomes is presented in Table 1.

**Table 1:** Clinical timeline: a case of Fournier’s gangrene and hairy cell leukemia A chronological summary of the patient’s clinical course, including symptom onset, diagnostic milestones, surgical interventions, and final outcome. This timeline highlights the rapid progression of infection and the temporal relationship with the diagnosis and treatment of hairy cell leukemia. ESBL: extended-spectrum beta-lactamase, HCL: hairy cell leukemia

Date	Event
Late February 2024	Initial onset of perianal pain and swelling
March 4, 2024	Surgical drainage of perianal abscess; ESBL-producing* E. coli *identified
March 8, 2024	Severe pancytopenia detected; bone marrow biopsy initiated
March 22, 2024	First dose of rituximab administered (HCL confirmed)
March 31, 2024	CT scan shows perirectal emphysema, abscess, and suspected rectal perforation
April 15, 2024	Surgery: colostomy, necrosectomy, and abdominal lavage
April 21, 2024	Patient died of septic shock despite ICU care

## Discussion

FG is a rapidly progressive and potentially fatal form of NF involving the perineal and genital regions. Although rare, it carries high morbidity and mortality, particularly in immunocompromised patients [3,7]. In this case, FG represented the initial presentation of previously undiagnosed HCL, a rare B-cell lymphoproliferative disorder that accounts for approximately 2% of adult leukemias [8,9].

HCL is characterized by pancytopenia, monocytopenia, and splenomegaly, leading to significant immunosuppression and a predisposition to severe infections [8-10]. Infectious complications may precede the hematologic diagnosis, as observed in our patient, in whom pancytopenia prompted further investigation and bone marrow biopsy. Notably, bacterial infections, especially those caused by Gram-negative organisms, have been reported as early manifestations of HCL [11].

The microbiological profile of FG is traditionally polymicrobial; however, monomicrobial infections due to *E. coli*, including ESBL-producing strains, are increasingly reported and represent a therapeutic challenge [3,7]. In our case, the isolation of an ESBL-producing *E. coli* necessitated escalation to carbapenem therapy, consistent with current recommendations for empiric management in high-risk patients [3].

Delayed diagnosis remains a major contributor to poor outcomes in FG. Imaging studies, particularly contrast-enhanced CT, play a pivotal role in assessing the extent of disease, identifying deep collections, and detecting complications such as bowel perforation [7]. In our patient, CT revealed a presacral abscess and rectal perforation, findings that prompted surgical exploration.

Surgical debridement is the cornerstone of FG management and should not be delayed, even in patients with profound cytopenias [11,12]. In our case, extensive necrosis and mesorectal involvement were identified intraoperatively. Although hemostasis was challenging due to thrombocytopenia, definitive surgical control was achieved. Studies have shown that patients with hematologic malignancy are significantly less likely to undergo timely surgery, contributing to increased mortality [11]

Several scoring systems, such as the Fournier’s Gangrene Severity Index (FGSI) and Uludağ FGSI (UFGSI), have been validated for mortality prediction in FG. These tools incorporate clinical and laboratory parameters and may support early decision-making. In our case, clinical deterioration and imaging findings were sufficient to justify aggressive intervention despite cytopenias.

Adjunctive therapies such as hyperbaric oxygen therapy (HBOT) or vacuum-assisted closure (VAC) have been proposed in select patients but remain controversial and were not utilized in this case [11,13]. In addition, initiating immunotherapy such as rituximab during active infection must be carefully individualized. Although rituximab was administered, the delayed initiation and progression of sepsis limited its impact [8,10].

This case illustrates the diagnostic and therapeutic complexity of FG in patients with occult hematologic malignancy. Clinicians should maintain a high index of suspicion for underlying immunosuppressive disorders in patients presenting with severe soft tissue infections and unexplained pancytopenia. Early multidisciplinary collaboration involving surgery, infectious disease, and hematology is essential to improve clinical outcomes.

## Conclusions

This case underscores the diagnostic and therapeutic challenges of NF in immunocompromised patients and highlights the potential for severe soft tissue infections to serve as the initial manifestation of an undiagnosed hematologic malignancy. Although typically indolent, HCL can present with profound pancytopenia and immunosuppression, increasing susceptibility to aggressive infections such as Fournier’s gangrene caused by multidrug-resistant organisms like ESBL-producing *E. coli*.

Despite appropriate antimicrobial therapy and surgical intervention, the outcome in this case was fatal, reflecting the synergistic effects of diagnostic delay, antimicrobial resistance, and impaired host immunity. Clinicians should maintain a high index of suspicion for underlying hematologic disease in patients with unexplained cytopenias and rapidly progressive infections unresponsive to standard therapy.

Timely hematologic evaluation, early imaging, and coordinated multidisciplinary management are critical to improving outcomes in this high-risk population. In addition, the rising prevalence of multidrug-resistant pathogens in necrotizing infections underscores the urgent need for targeted antimicrobial stewardship and further research into tailored therapeutic strategies for immunocompromised patients. Case reports such as this remain vital to raising awareness and advancing understanding of rare and life-threatening clinical presentations.
